# Glycohemoglobin A1c: A promising screening tool in gestational diabetes mellitus

**DOI:** 10.4103/0973-3930.45271

**Published:** 2008

**Authors:** Saleh A. Aldasouqi, David J. Solomon, Samia A. Bokhari, Patan M. Khan, Shareef Muneera, Ved V. Gossain

**Affiliations:** Department of Medicine, College of Human Medicine, Michigan State University, East Lansing, Michigan, USA; 1 Department of Medicine, King Fahd Armed Forces Hospital, Jeddah, Saudi Arabia

**Keywords:** A1c, diagnosis, gestational diabetes mellitus, HbA1c, screening

## Abstract

**CONTEXT:**

Current screening tests for gestational diabetes mellitus (GDM) are inconvenient. Therefore, alternative screening tests for GDM are desirable. The use of glycohemoglobin A1c (HbA1c) in screening for GDM remains controversial.

**AIM:**

We undertook this study to evaluate the utility of HbA1c in screening for GDM.

**SETTINGS AND DESIGN:**

Retrospective study in a tertiary teaching hospital.

**MATERIALS AND METHODS:**

Laboratory records were reviewed to identify pregnant women who underwent both oral glucose tolerance test (OGTT) and HbA1c measurements over a 16-months period. The association of OGTT with HbA1c was evaluated.

**STATISTICAL ANALYSIS USED:**

Data were collected using SPSS software. Comparisons of the means and calculations of sensitivities were performed.

**RESULTS:**

Of 145 eligible patients, 124 had GDM and 21 patients did not, per OGTT. The percentages of patients with HbA1c values (reference range of 4.8%–6.0%) equal to or above sequential cut-point values of 5.0%, 5.5%, 6.0%, 6.5% and 7.0% (i.e., sensitivity values) were 100%, 98.4%, 87.1%, 62.9% and 39.5%, respectively. The mean HbA1c of the patients with GDM was 6.9 + 0.8% compared to 6.4 + 0.6% for those without GDM (*P*< 0.006). At an arbitrary cut-off value of 6.0% (the upper limit of normal), HbA1c would have picked up 87.1% of patients with GDM.

**CONCLUSIONS:**

This study suggests that HbA1c is a reasonably sensitive screening measure of GDM in this high-risk population. Acknowledging limitations resulting from the study design, further prospective studies are warranted to verify this conclusion, and to evaluate the specificity of HbA1c as a screening test for GDM.

## Introduction

GDM is defined as glucose intolerance with onset or first recognition during pregnancy.[1,2] Screening for GDM has been controversial.[3] First proposed by O'Sullivan and Mahan over 3 decades ago,[4,5] screening for GDM is achieved by glucose challenge test (GCT) followed by oral glucose tolerance test (OGTT).[6,7] Some obstetricians utilize random blood sugar (RBS) in GDM screening.[8]

The OGTT is regarded as inconvenient and requires fasting, warranting the search for more convenient screening alternatives. Utilizing glycohemoglobin A1c (HbA1c) in GDM screening is controversial.[9–12] We undertook this study to evaluate HbA1c utility in GDM screening.

## Materials and Methods

This retrospective study was performed at King Fahd Armed Forces Teaching Hospital (KFAFH) in Jeddah, Saudi Arabia, and was approved by the KFAFH Research Ethics Committee. KFAFH is a tertiary teaching military hospital serving the military personnel and the civilian employees of the armed forces and their families within the Mid-Eastern Province of Saudi Arabia.

Antenatal care for KFAFH beneficiaries is provided by multiple primary care clinics within the hospital and the surrounding neighborhoods. As is the case in some other places, different screening methods are variably used in screening for GDM in these clinics. Both GCT and RBS are variably, but regularly, used. Universal GDM screening is adopted at all KFAFH clinics. For OGTT, the vast majority of physicians use the 3-hour 100-g OGTT recommended by the ADA,[6] and very few use the 2-hour 75-g test adopted by the World Health Organization.[7]

We utilized the hospital central laboratory database to identify the population of this study; this laboratory serves all the clinics, on- and off-campus. All OGTT tests that were performed between January 2001 and April 2002 were retrieved. Those tests associated with HbA1c measurements, performed concomitantly with OGTT or within two weeks of each other, were extracted. Corresponding patients were subsequently identified through an extensive review of medical records. To ensure homogeneity of data, we only included the 3-hour 100-g OGTT.

For the purpose of this study, the diagnosis of GDM was retrospectively based on the results of the OGTT tests, according to cut-off values recommended by the ADA guidelines.[6] These threshold values are: 5.3, 10.0, 8.6 and 7.8 mmol/L (95, 180, 155 and 140 mg/dl) for fasting, 1-, 2-, and 3-hours post-glucose load, respectively. Two or more abnormal values are needed for diagnosis of GDM.

Once all corresponding patients were identified and through a review of their medical records, we included in our analysis all consecutive pregnant women who had paired OGTT/HbA1c measurements performed during the third trimester. Because this was a retrospective study, we could not evaluate the selection criteria used for screening of these patients nor the screening method (GCT or RBS) used. However, it was assumed that screening was performed universally, according to the general trend of the primary care providers, as alluded to earlier. Furthermore, this study was not designed to evaluate the clinical course, management or outcome of the GDM in the study population.

The glucose measurements were performed on centrifuged venous blood, utilizing a standard commercial glucose oxidase method. The HbA1c assays were performed on EDTA whole blood, utilizing a Roche/Hitachi® analyzer (Tokyo, Japan). The assay method used in this analyzer is a turbidimetric inhibition immunoassay (TINIA). This assay method is not affected by either hemoglobinopathies or uremia.[13] The imprecision of the assay, co-efficient of variation percentages (% CV), were 1.8%-2.2% and 3.0%-4.4% for within run and between days, respectively.

The study data were collected manually and then input into a computer database by using an SPSS software program, version 10.0.0, for Windows (SPSS Inc., Chicago, IL, USA).

## Results

Of a total of 319 paired sets of OGTT-HbA1c results retrieved from the laboratory database, a total of 145 eligible subjects were identified upon review of medical records. Of those patients, a total of 124 had a confirmed diagnosis of GDM according to the OGTT results. These patients were all Saudis with the exception of two Philippinos and one Yemeni. Their age (years), weight (kg) and gestational age (weeks) were 33 ± 6, 81 ± 16 and 32 ± 5 (Mean ± SD), respectively [Table 1].

**Table 1:** Demographical data and results of HA1c results as compared to OGTT, with sensitivities of HbA1c at graded values around 6.0%

Number of patients	145
Number of patients with GDM (Positive OGTT)	124
Number of patients without GDM (Negative OGTT)	21
Age (years)*	33 ± 6
Weight (kg)*	81 ± 16
Gestational age (weeks)*	32 ± 5
Mean A1c (%) for patients with GDM	6.9 ± 0.8
Mean A1c (%) for patients without GDM	6.4 ± 0.6 (p < 0.006)
Sensitivity (%) of HbA1 at cut-point value of the following:	
≥ 5.0	100
≥ 5.5	98.4
≥ 6.0	87.1
≥ 6.5	62.9
≥ 7.0	39.5

Results are presented as Mean ± SD, HbA1c: Hemoglobin A1c, OGTT: Oral glucose tolerance test.

The screening methods that could be identified from reviewing the medical records of the study patients (*n* = 145) were as follows: RBS (n=81, 67 with and 14 without GDM according to OGTT results); GCT (*n* = 29, 27 with and 2 without GDM). One patient had both tests performed. In the remaining patients (*n* = 36), we could not identify the screening method(s) employed upon an extensive review of laboratory databases and medical records.

The local laboratory reference range for HbA1c was 4.8%–6.0% for the general population. The percentages of patients with diagnosed GDM who had HbA1c values above or equal to the upper reference cut-point values of 5.0%, 5.5%, 6.0%, 6.5% and 7.0% were100%, 98.4%, 87.1%, 62.9% and 39.5%, respectively [Table 1]. As compared to the mean HbA1c of 6.9 ± 0.8% of those patients with GDM (*n* = 124), the HbA1c of the 21 patients with normal OGTT results was 6.4 ± 0.6% (*P* < 0.006).

## Discussion

This study has shown that the majority of women (108 of 124 or 87.1%) with confirmed diagnosis of GDM had elevated HbA1c (≥ 6.0%, the upper limit of normal according to the local laboratory). This suggests a reasonable sensitivity of HbA1c as a predictor of OGTT (the diagnostic gold standard), at an arbitrary HbA1c cut-point of 6%; due lack of controls, our data did not allow for a meaningful calculation of the test specificity. The study has also shown that the average HbA1c of the 21 patients with normal (negative) OGTT results (without GDM) was 6.4 ± 0.6% (*P* < 0.006). Of these 21 patients, 16 (76.2%) had HbA1c ≥ 6.0%.

Because of these (apparent) many false positives, which may suggest a lower specificity, we further analyzed the OGTT results of the aforementioned patients (*n* = 21) with normal (negative) OGTT results [Table 2]. Of these 21 patients, 16 had HbA1c ≥ 6.0%, and 5 had HbA1c < 6.0%. Of the 16 patients with HbA1c ≥ 6.0%, 13 (82%) had OGTT results that did not qualify as positive (diagnostic of GDM) but were in between normal glucose tolerance (NGT) and GDM.

**Table 2:** Results of HbA1c and OGTT values for patients with OGTT results, which are considered normal (negative) for GDM according to OGTT

No.	HA1c (%)	0 hour*	1 hour	2 hours	3 hours
1.	5.8	4.8	9.8	8.2	4.9
2.	6.9	3.8	8.5	6.1	6.2
3.	7.2	4.3	9.1	8.8	7.0
4.	6.5	4.5	9.0	7.1	7.1
5.	5.9	4.9	8.5	5.7	6.2
6.	6.3	4.3	9.4	8.3	**
7.	6.3	4.8	9.6	6.3	**
8.	6.2	4.4	6.3	5.2	4.1
9.	6.5	4.4	9.8	7.3	5.8
10.	6.3	4.9	9.8	10.2	**
11.	6.3	5.2	8.9	8.2	**
12.	5.9	4.7	9.6	8.0	5.8
13.	6.9	5.1	7.6	6.4	5.9
14.	6.2	5.2	7.9	5.9	3.8
15.	7.0	4.3	8.3	7.1	8.1
16.	6.5	4.5	8.8	7.9	4.2
17.	6.2	4.9	9.5	7.9	**
18.	7.0	4.2	5.5	4.8	6.3
19.	5.0	4.6	9.2	7.9	6.7
20.	5.4	4.6	9.2	8.3	7.5
21.	7.9	5.1	10.7	8.2	5.6

* Glucose values are expressed here in mmol/L (to covert to mg/dl, the values are multiplied by 18). The data were extracted from the original database, as our laboratory use the SI units. The OGTT cut-off thresholds for ADA diagnostic criteria are 5.3, 10.0, 8.6 and 7.8 mmol/L for fasting, 1-, 2- and 3-hours post glucose load.

** Missed results due to patients not returning for the 3rd hour glucose test.

Of these 13 patient with borderline OGTT results short of GDM diagnosis, 11 had one or more glucose values that were very close to the diagnostic cut-off thresholds, and 4 had one abnormal glucose value each; three patient had one of each [Table 2]. Thus, only three of these 16 patient had unequivocal NGT by OGTT. Therefore, it is conceivable that the elevated HbA1c in the majority of these 21 presumably non-GDM patient is truly abnormal, reflecting mild mean hyperglycemia.

This situation of intermediate glycemia (or mild hyperglycemia) has been recently addressed. Cianni *et al.* have reported[14] that pregnant women with OGTT results short of GDM diagnosis are distinct from those with NGT, and should not be treated as normal. They reported that these patients had insulin resistance and relative insulin deficiency and may be at risk for the usual GDM complications (e.g., macrosomia). The authors[14] labeled this condition as one abnormal value (OAV).

Of note, the aforementioned arbitrary upper-limit cut-off value (6.0%) which we used in the study is generally applicable in nonpregnant populations. However, a close cut-off value has been recently validated for pregnant women. Balaji *et al*.[15] have recently published normal mean HbA1c values in Asian Indian pregnant women as 5.36 ± 0.36%. Deriving a reference range (mean ± 2 SD) of 4.64–6.08 from these data, the resulting cut-off value, 6.08%, is reasonably comparable to ours.

Nevertheless and until a known cut-off value is universally adopted, we included results of sensitivities across a graded spectrum of five HbA1c cut-off values of 0.5% around the 6.0% cut-off, i.e., from 5.0% to 7.0%. These results were, expectedly dropping from 100% for the 5.0% cut-off value to 39.5% for the 7.0% value. These dropping sensitivity values are typical of diagnostic tests, losing sensitivity towards the higher-end values [Table 1]. Our data did not allow enough data for reasonable calculation of specificity, and therefore, we opted not to include an ROC curve, which would be desirable herein.

Our findings thus suggest a potential screening role for HbA1c, as a predictor of abnormal OGTT in this high risk population. In other words, if HbA1c was used as a screening measure in these patients, using the arbitrary cut-point of 6.0%, only 12.9% of the patients with confirmed GDM would be missed (i.e., up to 87.1% would be picked up).

Pioneered by Pollack and associates,[9] previous studies addressing the diagnostic potential of HbA1c in GDM were discrepant and they lacked consensus.[9–12] In a recent publication, Agarwal and associates reported that all prior studies addressing this issue had not been recent, long before the era of HbA1c standardization.[11] Various and less advanced HbA1c assays were used in those studies, and they produced conflicting conclusions.[11]

The most recently published studies addressing the screening potential of HbA1c in GDM were both reported by the same investigators (Agarwal and associates), within the last six years.[10,11] It is interesting to note that, similar to older studies, the authors of these two recent studies reported discrepant conclusions regarding specificity and sensitivity of HA1c as a GDM screening tool. Therefore, it seems that debate continues in the issue of HbA1c validity for GDM screening.

Our study has obvious limitations. First of all, the study was designed as a retrospective study, involving a special high-risk population. As such, the number of subjects with normal OGTT was too small to allow for calculation of the test specificity or negative predictive values. Since we are looking at HbA1c test solely as a screening test in this study, we believe that sensitivity is more relevant than specificity.

Second, the study did not evaluate the clinical status or outcome of the subjects or how the screening methods were allocated as explained earlier. Using different screening methods added heterogeneity to our data. Finally, our study was not designed to evaluate the HbA1c level in relation to fetal outcomes, such as birth weight or neonatal complications. These clinical implications would be desirable in studies involving diagnostic tests.

In conclusion, this study suggests that HbA1c may be a reasonably sensitive screening measure for prediction of GDM in high risk population. In other words, we suggest that HbA1c be used as an adjunct to GCT or RBS in screening for GDM in these populations. Further studies are warranted to evaluate the specificity and other diagnostic parameters of HbA1c before endorsing it as an alternative screening tool in populations.
